# Volumetric Modulated Arc Therapy (VMAT): A modern radiotherapy technique - A single institutional experience

**DOI:** 10.12669/pjms.37.2.2647

**Published:** 2021

**Authors:** Azhar Rashid, Zaeem Ahmad, Muhammad Ali Memon, Abdul Sattar M Hashim

**Affiliations:** 1 Dr. Azhar Rashid, MBBS, FCPS, MSc. Clinical & Radiation Oncologist, Department of Radiation Oncology (Stereotactic Radiosurgery), Neurospinal & Cancer Care Institute (NCCI), M.A Jinnah Road, Saddar, Karachi, Pakistan; 2 Mr. Zaeem Ahmed, MSc Physics Medical Physicist, Department of Radiation Oncology (Stereotactic Radiosurgery), Neurospinal & Cancer Care Institute (NCCI), M.A Jinnah Road, Saddar, Karachi, Pakistan; 3 Dr. Muhammad Ali Memom, MBBS, FCPS. Clinical & Radiation Oncologist, Department of Radiation Oncology (Stereotactic Radiosurgery), Neurospinal & Cancer Care Institute (NCCI), M.A Jinnah Road, Saddar, Karachi, Pakistan; 4 Dr. Abdul Sattar M Hashim, MBBS, MD, PhD. Chief Neurosurgeon, Department of Stereotactic Radiosurgery/Neurosurgery, Neurospinal & Cancer Care Institute (NCCI), M.A Jinnah Road, Saddar, Karachi, Pakistan

**Keywords:** Conformity Index, Homogeneity Index, Radiotherapy, VMAT

## Abstract

**Objective:**

To evaluate VMAT plans for conformity and homogeneity of radiation dose to the target in order to share our experience as a pioneering institute to use VMAT technology in Pakistan.

**Methods:**

Since December 2014 to January 2018, 530 patients of various anatomical sites were treated by VMAT technique at Neurospinal Cancer Care Institute (NCCI) Karachi Pakistan. ERGO++ planning system (Version 1.7.2) was used to develop VMAT plans with single or multiple arcs by the rotation of couch and gantry. The plans were evaluated by calculating Conformity Index (CI) and Homogeneity Index (HI) and critical organ (OARs) doses of individual tumor sites.

**Results:**

The average CI of various sites was 1.4 (range: 1.0–2.0) and average HI of various sites was 1.20 (range: 1.07–1.374), respective critical organ doses were adequately achieved.

**Conclusions:**

VMAT treatment planning technique showed good conformal and homogeneous target coverage with sparing of organs at risk and reduced treatment delivery time. With these features, safety of VMAT technique may allow its routine clinical use, though it is still under investigation in many areas.

**Abbreviations Used**
CI: Conformity Index
HI: Homogeneity Index
GI: Gradient Index
SIB: Simultaneous Integrated Boost
SRS: Stereotactic Radiosurgery
SBRT: Stereotactic Body Radiotherapy
VMAT: Volumetric Modulated Arc Therapy
MLCs: Multi-leaf Collimators
MU: Monitor Units
DAM: Dynamic Arc module
TV: Target Volume
MD: Maximum Dose
PD: Prescription Dose
RTOG: Radiation Therapy Oncology Group
Linac: Linear accelerator

## INTRODUCTION

Significant advancement has occurred in the radiotherapy techniques and deliveries during the past few decades worldwide. The main goal is to achieve highly conformal dose distributions and to improve the therapeutic ratio.^1^

In 1965, Takahashi was the first to describe arc therapy using dynamic field shaping using MLCs.^2^ In the late 1990s, intensity-modulated radiation therapy (IMRT) was introduced and soon adopted in clinical practice. IMRT is generally a broad term to define the different types of advanced radiotherapy treatment deliveries also including the arc therapy.^3^,^4^ Yu in 1995, was the first one to give the concept of Intensity modulation Arc Therapy (IMAT) in which continuous gantry rotation and dynamic MLC motion combine to produce modulation during the beam on time.^5^ Several arcs were used with different gantry angles along with the segments as the approach of classical IMAT.

Volumetric Modulated Arc Therapy (VMAT) was introduced in 2007 and was described as a novel radiation technique that has continuous modulation of MLCs (field shaping), along with the dose rate and gantry speed rotation utilized to deliver highly conformal dose distributions simultaneously, in a minimal time period with reasonable MUs to be delivered.^6^,^7^ Otto^8^ did a remarkable job by developing the treatment planning algorithm for single arc VMAT. VMAT carries an advantage over IMAT in terms of greater degree of freedom with above factors that increase the capability of beam intensity modulation. Teoh et al.^6^, published comprehensive review article on VMAT in 2011. VMAT is becoming an increasingly utilized radiation technique. It proved as a novel and emerging technology with freedom of selection of number of arcs and some other features making it more efficient and faster in terms of treatment time and MUs delivery, its ability to spread low dose to a wide area of normal tissue, ability to deliver complex treatments with coplanar or non-coplanar single or multiple arcs make it a unique technology. The risk of secondary malignancy in VMAT should be lower as VMAT generally uses fewer monitor units (MU) compared with conventional fixed field IMRT. Macchia et al.^1^, published a detailed review of VMAT and its clinical use for various body sites in 2017, this study described that VMAT has been used mostly for brain tumors, head & neck, thoracic cancers, GU cancers, GI cancers and SBRT for oligometastasis.

VMAT-SIB is a popular technique that allows treatment of several volumes with different dose prescriptions called simultaneous integrated boost (SIB) is conveniently executed with VMAT technology that results into delivery of high biological effective doses to the target and reduction of the dose to the surrounding normal tissues and improvement of the toxicity. Macchia et al.^1^, concluded that the clinical use of VMAT is less documented, but VMAT-SIB and VMAT-SBRT is an effective and safe technique for various cancers of the body.^1^ More clinical data will emerge by the time as the numbers of patients are increasing across the world.

VMAT-SRS is described by Hanna et al.^9^, as a reliable therapeutic modality of SRS based upon the existing dosimetric research on its safety and benefits particularly in multiple brain metastases. They considered VMAT similar to the “non-VMAT” approach in terms of treatment plan acceptability (conformity and heterogeneity), treating multiple lesions and offering frameless radiosurgery treatments under image guidance.

Dr. Shahid Hameed (Radiation Oncologist) is the pioneer of modern radiotherapy in Pakistan, who started IMRT treatments in 2005 at Shaukat Khanum Memorial Cancer Hospital and Research Center (SKMCH & RC). SKMCH & RC Lahore is the first institution where modern radiotherapy was developed.^10^ Later in 2008, Gamma knife & Linac based Stereotactic Radiosurgery/Radiotherapy setup was established at Neurospinal & Cancer Care Institute (NCCI) Karachi by Prof. A. Sattar M Hashim (Neurosurgeon). So, our group emerged as a pioneer of stereotactic radiosurgery (SRS) /stereotactic body radiotherapy (SBRT) in Pakistan. Initially we have been using various forms of IMRT: fixed beam, step and shoot, forward and inverse IMRT from 2008 till 2013. Later we were able to have a license to use VMAT technology. Since 2014 we are using VMAT, VMAT-SIB and VMAT-SRS/SBRT techniques on regular basis. In present work, we report our institutional experience of using VMAT treatment technique in Pakistan.

## METHODS

From December 2014- January 2018, 530 patients were treated with VMAT technique. This study was carried out at Neurospinal Cancer Care Institute (NCCI) Karachi Pakistan. The study was approved from the hospital Ethics Committee/IRB on 01.04.2020 (# NCCI/IRB/2020/01).

### Patients

Patient work flow for VMAT is similar to regular IMRT/Stereotactic Radiosurgery technique. It has been described in our previous publication 11. We use to have PET-CT scanner (SIEMENS) simulations preferably for lung, esophagus, recurrent and metastatic disease. MRI (TOSHIBA, 1.5 Tesla) was additionally done for brain, head & neck and pelvic malignancies.

### Volume Delineation

**Fig.1:**
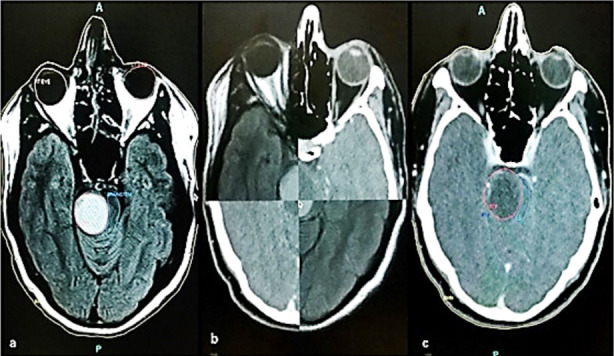
(a): Contouring of GTV and remaining brainstem on MR images. (b): Fusion of MRI & CT images. (c): After fusion, contouring shifted to CT images for planning.

Target location and organs contouring is an important component. In most of the cases, image fusion between CT and MRI images was done for better localization and optimization (Fig.1). Planning target volume (PTV), Clinical target volume (CTV), Gross tumor volume (GTV) and planning organ at risk volume (PRV), other organs at risks (OARs) were delineated by the radiation oncologist with the help of the Radiologist (when needed).

### Linear accelerator

The linear accelerator used for the delivery of VMAT was Elekta Synergy-S, having photon energy of 6MV. It has beam modulator head comprised of micro-multileaf collimators (mic-MLCs) of width 4mm with 40 leaf pairs and maximum field size of 21cm x 16cm with no moveable jaws and the fixed diaphragms. Minimum MU per degree rotation is 0.10 MU/ degree and maximum gantry speed is 6º/sec. The maximum speed for leaf is around 2.5cm/sec. The actual dose rate for the VMAT delivery is 500 MU/min which can be differ by ±10%. The Synergy-S is controlled with the Elekta Integrity system version 1.1 which automatically selects the combination of dose rate, gantry speed and MLCs speed during the VMAT technique.

### Treatment planning

The plans were constructed with the version 1.7.2 of ERGO++ treatment planning system in dynamic arc module (DAM) which is capable of doing the VMAT planning. Single / multiple arcs were used. The step size for all single and multiple arcs used was of 10^°^ between two gantry angles. The arc angles were selected manually with the same step size of 10º. So, a 10º step size is referred to as a beam. Collimator angle is defined for each arc according to the conformity of the tumor. Gantry rotation speed, MLCs position and dose rate were dependent on the optimization.

**Fig.2:**
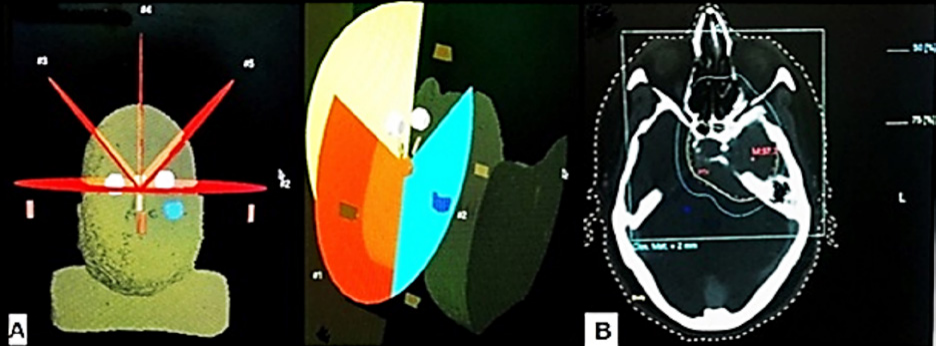
(a): Crown technique used for brain tumor with different couch angles. (b): Prescription was made on 75% isodose line(green) covering peripheral part of PTV, while blue line represents 50% isodose line.

**Fig.3:**
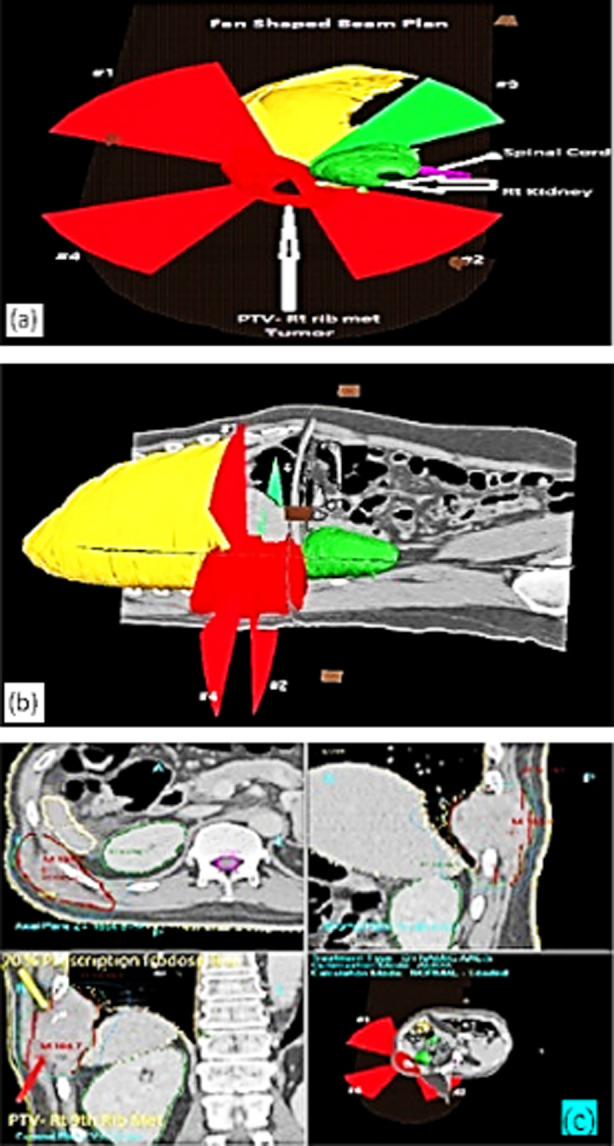
VMAT-SBRT, Fan shaped beam planning display: (a) Right rib metastases was planned for 10 Gy x 3 Fr @ 70% isodose line. (b) Planning with CT images (c) Three dimensional view.

**Fig.4:**
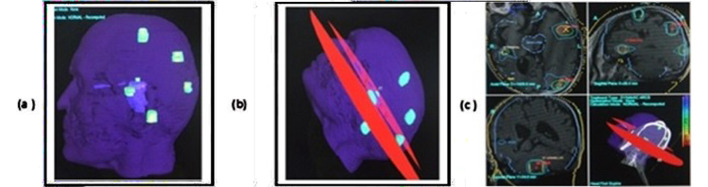
VMAT-SRS mono-isocentric planning display for multiple (six) brain metastases from non-small cell lung cancer. Dose prescription was 20 Gy single fraction at 70% isodose line. a) Location and Volume of Six brain metastases, Left temporal met: 1.8 cc, Left parietal met: 2.1 cc, left posterior parietal met: 2.2 cc, Left Occipital met: 1.2 cc, Right parietal met: 0.3cc, Right cerebellar met: 2.9cc. b) Plan was prepared with the help of two arcs, all the contoured metastases were well covered with prescription isodose line of 70%. Total treatment time was 11 minutes. c) Computerized planning on treatment planning system ERGO++. Green line around the target is prescription isodose line of 70%. Blue line is 50% isodose line.

In most of the cases, single arc was used. Single arc sometimes does not lead to better plan quality, however different arcs were used for the better coverage of the tumor along with the minimum dose to the critical organs as described by Guckenberger et al.^12^ In some cases for better conformity and sparing of normal organs, Crown shaped arcs were used in which couch rotation with different angles were used (Fig.2). Few cases were treated with splitted arcs like a fan beam shaped to save the spinal cord in esophageal cancer. There is fan shaped planning display for VMAT-SBRT in right rib metastases (Fig.3). While VMAT-SRS planning in multiple brain metastases is shown in (Fig.4).

### Plan Evaluation

The clinical and technical acceptability of the plans were evaluated by the radiation oncologist and the physicist team. The evaluation is based upon the dosimetric quantities for the PTV, CTV, GTV and OARs. The dose volume histograms (DVH) and isodose curves were the main tools to use for the plan evaluation. Each contoured target or organs were evaluated by both the integral and differential DVHs. We used RTOG-1993 criteria for assessment of plans to calculate Conformity Index (CI) and Homogeneity Index (HI).^13^

*Conformity Index (CI):* It is defined as the ratio of the prescription isodose volume (PI) divided by the target volume (TV). Following formula was used:

**CI =** prescription isodose volume (PI) / target volume **(TV).** If this ratio was between 1.0 and 2.0, treatment plan was acceptable.

*Homogeneity Index (HI):* is defined as the ratio of the maximum dose (MD) divided by the prescription dose (PD). Following formula was used:

**HI =** maximum dose (MD) / prescription dose **(PD)**

If this ratio was less than or equal to two, treatment plan was acceptable.

Critical organ (OARs) doses were evaluated as given by Emami et al. and Timmerman RD.^14^ Finally plan was approved after above evaluations; however, plan was re-checked by another medical physicist before transferring it to the MOSAIQ.

### VMAT treatment

The MOSAIQ (Record and Verify) system is connected to Integrity system attached with the Synergy- S, operates the linac control system automatically. A test run was performed before the treatment of all the patients so as to check the possible errors during the delivery like the motion of MLCs, gantry rotation, and fluent dose delivery without any obstacles.

### Setup verification

Patient position and setup for radiation dose delivery contribute a major part in every radiotherapy technique. Cone Beam CT (CBCT) mounted on our linear accelerator and a software X-ray volumetric imaging system (XVI) is available for image guidance applications. It operates in kilo-voltages and has scan duration of 30 – 50 seconds approximately. The scan is with the gantry rotation of 100° to 180° depending on the scans of different tumor sites providing 3-D images with the coordinates to match with the reference image. On-line set-up errors are determined in a two-step process. First the XVI data is registered to the planning data by matching the machine-isocenter with the planning-isocenter, respectively. The machine isocenter is defined in the XVI data during the reconstruction. The planning-isocenter is defined during the planning process in the planning CT data. Set-up errors are then determined from a second registration to remove residual displacements.

## RESULTS

The average CI of various sites was 1.4 (range: 1.0–2.0) and average HI of various sites was 1.20 (range: 1.07–1.374), critical organ (OAR) doses in respective region were adequately achieved as given by Emami et al. and Timmerman RD.^14^ Most of the patients 381(72%) were of brain tumors (Primary & Metastases) with average CI of 1.1 and average HI of 1.141, while the doses of critical organs like optic chiasma, optic nerves, brain stem were adequately achieved. Head & neck cases were 43 (8%) with average CI of 1.6 and average HI of 1.271.Liver cases were the next in line with 42(8%) patients having average CI of 1 and average HI of 1.374. Esophageal cancer cases were 21( 4%) with average CI of 1.6 and average HI of 1.1. Lung tumors were 16(03%) with average CI of 1.5 and average HI of 1.215. Spine cases were 11(02%) with average CI of 1.5 and average HI of 1.364. Rectal cancer cases were 11 ( 02%) with average CI of 2.0 and average HI of 1.085.Prostate cancer cases were 05(01%) with average CI of 1.2 and average HI of 1.07. Results have been summarized in Table-I, showing the treated sites, Conformity Index, Homogeneity Index and treatment volumes.

**Table-I:** Treated sites with average Conformity Index (CI), average Homogeneity Index (HI), and treatment volumes.

*Treated site*	*No. of patients*	*Percentage*	*Average CI*	*Average HI*	*Average planning target volume (cc)*
Brain	381	72%	1.1	1.141	85.08
Head & Neck	43	08%	1.6	1.271	361.34
Liver	42	08%	1.0	1.374	33
Esophagus	21	04%	1.6	1.1	440
Lung	16	03%	1.5	1.215	251.1
Spine	11	02%	1.5	1.364	207.18
Rectum	11	02%	2.0	1.085	646.78
Prostate	05	01%	1.2	1.07	186.3
Total Number of Patients(N)	530	100%	1.4 (range:1.0-2.0)	1.20 (range:1.07-1.374)	276.34 (range: 33-646.78)

## DISCUSSION

To assess the plan quality, many tools are available and we chose CI and HI to document the acceptance of plans for VMAT. Feuvret L et al.^15^, described CI as a complementary tool that can be used to score or compare several treatment plans for the same patient.

Kataria T et al.,^16^ used Homogeneity Index (HI) to find out the co-relation between HI and prescribed dose, target volume and target location. There was no significant co-relation between the location and volume of target but there was a trend toward better HI with increasing prescribed dose. Comparisons were made between VMAT and IMRT or other advance radiotherapy techniques in published literature and VMAT was found to be a beneficial either in the form of escalation of dose to the target, dose reduction to the critical organs, reduced treatment time or better conformity.

In current study, we rationalize to use VMAT for brain (Primary & Mets), head & neck, liver, esophagus, lung, spine, rectum and prostate and found comparable results in terms of conformity, homogeneity and critical organ doses.

One of the studies by A Atiq et al.^17^, chose 13 cervical cancer patients treatment plans for dosimetric comparison between RapidArc and IMRT. They found RapidArc has better conformity and homogeneity along with high dose gradient, improved dosimetry and treatment efficiency in comparison to IMRT. This study revealed that Average CI for RapidArc was 1.59 and for IMRT it was 1.02, while average CI for all the sites in our study is 1.4. Whereas average HI for RapidArc was 1.12 and for IMRT it was 1.13 and we have an average HI of all the body sites1.2.

Khan et al.^18^, studied Prostate cancer patients for IMRT and VMAT. They analyzed 90 patients out of which forty patients for IMRT and fifty patients for VMAT were enrolled in the study. They found comparable doses for both the techniques and VMAT was validated based on dosimetric and radiobiologic outcomes. Moreover, increased number of patients were treated because of reduced treatment time and durability of the linear accelerator was improved because of lesser MU used in VMAT.

Kim et al.,^19^ studied 26 patients of primary brain tumor and found sparing of contralateral hippocampus when treated by VMAT for verbal memory function preservation. Sheu et al.^20^, recommended VMAT as a potential radiation modality for treatment of GBM patients. They conducted a comparative study on 88 diagnosed patients of GBM. 45 patients were treated with IMRT and 43 patients were treated with VMAT. Both the groups had similar survivals and toxicity. The mean time of treatment in VMAT was reduced by 29%. In VMAT, mean time of treatment was 10.3 minutes and it was 14.6 minutes for IMRT. Time of treatment was statistically significant as P-value was < 0.01. This shorter treatment time may improve resource utilization.

Li et al.^21^, presented comparison between RapidArc and Helical Tomotherapy for early T-stage Nasopharyngeal carcinoma. They concluded that RapidArc is better in delivering lesser MUs so shorter treatment times, also better sparing of optic nerve and optic chiasma. They also found that dose homogeneity, D98, and sparing of spinal cord and pituitary is also better in Rapid Arc.

In a study conducted by Ruggieri et al.^22^, 20 patients were presented. Mono Isocentric (HyperArc) vs multiple isocentric VMAT were compared for multiple brain metastases. They found mono isocenteric plans having higher CI and a lower gradient Index (GI) than standard multiple-isocenter VMAT plans, there was reduction in V12 to the brain-minus- PTV and significant treatment time reduction was observed.

Zhang et al.^23^, compared 15 patients of multiple brain metastases for VMAT vs cyberknife. They concluded that VMAT had less MUs and shorter beam on time than cyberknife while both have comparable dose falloff at high dose levels.

Above literature revealed that both the plan quality and treatment time indicates the clear advantage of VMAT over other radiotherapy techniques. Improved Conformity Indices and Homogeneity indices with comparable critical organ doses with IMRT and other treatment techniques, shorter treatment time and safe delivery favors VMAT technique to adopt in a regular clinical work flow. Short treatment time is an important factor especially for patients who have pain and move during treatment, can have single arc VMAT technique reducing the treatment time to 35 to 40%.^24^ This reduction in treatment time is much important for the patient comfort and for the departments having huge workload. The radiotherapy appointment times can be reduced to accommodate more number of patients.

### Limitation of this study

Its design is retrospective. No comparative analysis presented.

This manuscript is an addition to the local literature of cancer patient treatment with radiotherapy. It explains modern radiotherapy technique which has unique characteristics. This study will guide the new users in the country to adopt this beneficial modern technique as quickly as possible for the best interest of patients and radiation oncology departments.

## CONCLUSION

VMAT treatment planning technique showed good conformal and homogeneous target coverage with sparing of organs at risk and reduced treatment delivery time. With these features, safety of VMAT technique may allow its routine clinical use, though it is still under investigation in many areas.

### Author`s Contribution:

**AR** conceived, designed, writing and editing of manuscript.

**ZA** did data collection and manuscript writing.

**MAM and ASMH** participated in writing & editing of manuscript.

**AR** takes the responsibility and is accountable for all aspects of the work in ensuring that questions related to the accuracy or integrity of any part of the work are appropriately investigated and resolved.
